# Mitochondrial dysfunction triggers Zbp1-mediated necroptosis and inflammation in acute lung injury

**DOI:** 10.17305/bb.2025.13046

**Published:** 2025-10-29

**Authors:** Mi Zhou, Yuehan Li, Yinying Ren, Yan Li, JinYing Xiang, Fang Deng, Gang Geng, Jian Luo, Jinyue Yu, Zhou Fu, Fengxia Ding, Bo Liu

**Affiliations:** 1 Department of Pediatric Respiratory Medicine, National Clinical Research Center for Child Health and Disorders, Ministry of Education Key Laboratory of Child Development and Disorders, China International Science and Technology Cooperation Base of Child Development and Critical Disorders, Chongqing Engineering Research Center of Stem Cell Therapy, Children’s Hospital of Chongqing Medical University, Chongqing, China; 2 Bristol Medical School, University of Bristol, Bristol, U.K; 3 Great Ormond Street Institute of Child Health, University College London, London, U.K; 4 Department of Pediatric Cardiothoracic Surgery, National Clinical Research Center for Child Health and Disorders, Ministry of Education Key Laboratory of Child Development and Disorders, China International Science and Technology Cooperation Base of Child Development and Critical Disorders, Chongqing Engineering Research Center of Stem Cell Therapy, Children’s Hospital of Chongqing Medical University, Chongqing, China

**Keywords:** Acute lung injury, alveolar macrophages, mitochondria, Zbp1

## Abstract

Acute lung injury (ALI) is driven by dysregulated inflammation, but how mitochondrial damage engages necroptosis in alveolar macrophages (AMs) remains unclear. We aimed to define the mechanistic link between mitochondrial impairment and Zinc finger protein 1 (Zbp1)-mediated necroptosis in the murine AM-like cell line (MH-S). MH-S cells were stimulated with lipopolysaccharide (LPS) and profiled by RNA sequencing; necroptotic death was quantified by Calcein-AM/propidium iodide staining and lactate dehydrogenase release, Zbp1 localization was examined by immunofluorescence microscopy, and Zbp1, receptor-interacting protein kinase 3 (RIPK3)/phospho-RIPK3 (p-RIPK3) and mixed lineage kinase domain-like protein (MLKL)/phospho-MLKL (p-MLKL) were measured by Western blotting. Mitochondrial status was assessed by mitochondrial reactive oxygen species (mtROS), mitochondrial membrane potential (Δ Ψm; JC-1), mitochondrial permeability transition pore opening, adenosine triphosphate (ATP) content, and the markers ATP synthase F1 subunit alpha (ATP5a1), mitochondrial transcription factor A (TFAM), and translocase of outer mitochondrial membrane 20; inflammatory responses were quantified by flow cytometry and qPCR. The mitochondria-targeted antioxidant Mito-TEMPO (MT) was used to interrogate the role of oxidative stress. LPS markedly increased Zbp1 transcription, coincident with upregulation of pro-inflammatory genes and activation of necroptosis; mitochondrial damage and elevated mtROS were critical upstream events for Zbp1 induction, driving RIPK3 and MLKL phosphorylation, necroptosis, and cytokine release. MT restored mitochondrial function, lowered mtROS, downregulated Zbp1 and its necroptotic effectors (p-RIPK3, p-MLKL), and significantly reduced both necroptotic injury and inflammatory output. Collectively, mitochondrial dysfunction-driven mtROS initiates the Zbp1/RIPK3/MLKL necroptotic axis in AMs, thereby amplifying pulmonary inflammation in ALI; targeting mtROS may mitigate necroptosis and protect against lung injury.

## Introduction

Acute lung injury (ALI) is a critical clinical syndrome characterized by extensive damage to the alveolar epithelium and heightened, poorly controlled inflammation, contributing to high morbidity and mortality rates globally [1–3]. Although multiple pathological factors contribute to its development, a central hallmark of ALI is immune dysregulation, which drives the progression of inflammatory injury within the lungs [4]. Alveolar macrophages (AMs), the sentinel immune cells of the pulmonary microenvironment, play a crucial role in detecting tissue damage and orchestrating immune responses [5]. However, the exact mechanisms by which AM dysfunction leads to exacerbated inflammation in ALI remain inadequately characterized.

Mitochondria, traditionally known for their role in energy metabolism, also function as critical hubs for maintaining cellular integrity, regulating redox balance, and modulating programmed cell death [6, 7]. Recent evidence has identified mitochondrial derangement as a significant contributor to inflammation and tissue injury across various disease contexts, including ALI [8]. Notably, mitochondrial quality control (MQC) systems and cell death pathways are closely intertwined and essential for maintaining lung homeostasis [9–12]. Disruption of MQC, alterations in mitochondrial dynamics, and compromised membrane integrity can lead to oxidative stress and the release of damage-associated molecular patterns (DAMPs), thereby amplifying inflammatory signaling cascades [13]. Furthermore, mitochondrial stress has been linked to the activation of necroptosis, a form of regulated necrosis characterized by loss of membrane integrity and pro-inflammatory cellular disintegration [14].

Necroptosis is mediated by a well-defined signaling cascade involving receptor-interacting protein kinases RIPK1 and RIPK3, which activate mixed lineage kinase domain-like protein (MLKL) through phosphorylation. This activation leads to MLKL oligomerization and subsequent disruption of the plasma membrane [15]. Unlike apoptosis, necroptosis occurs independently of caspases and induces robust inflammation, serving as a crucial link between cell death and immune activation in various inflammatory conditions [16]. Recent research has identified Zinc finger protein 1 (Zbp1), a cytosolic RHIM-containing sensor, as an upstream activator of receptor-interacting protein kinase 3 (RIPK3)-dependent necroptosis [17]. Although Zbp1 is implicated in antiviral responses and chronic inflammation, its role in ALI, particularly in the context of mitochondrial damage, remains poorly understood.

Given the critical function of AMs in regulating pulmonary inflammation during ALI, it is essential to investigate how their mitochondrial integrity influences Zbp1-driven necroptotic signaling. This study aims to elucidate the interplay between mitochondrial impairment in AMs and the initiation of Zbp1-mediated necroptosis in the pathogenesis of ALI. Understanding these molecular interactions may provide novel mechanistic insights and potential therapeutic strategies to mitigate inflammatory lung injury.

## Materials and methods

### Transcriptome profiling of MH-S cells treated with lipopolysaccharide (LPS)

The murine AM-like MH-S cell line (5 × 10^6^ cells/group) was cultured under both baseline and LPS-stimulated conditions. Total RNA was extracted using 1 mL of TRIzol reagent (TIANGEN, China). The extracted RNA was used for library preparation and sequencing, conducted by Shanghai Biotechnology Corporation. Complementary DNA (cDNA) libraries were constructed following the TruSeq^®^ RNA Sample Preparation guidelines, and final libraries were generated through PCR-based amplification.

After purification, library quality and concentration were assessed. Sequencing was performed using the Illumina HiSeq 2500 platform (Illumina, USA). Raw reads were processed to obtain transcriptomic profiles. Differentially expressed genes (DEGs) were identified using the edgeR statistical package, with significance criteria set at |log_2_ fold change | ≥ 1 and adjusted *P* value < 0.05. To explore the biological implications of LPS exposure in MH-S cells, Gene Ontology (GO) and Kyoto Encyclopedia of Genes and Genomes (KEGG) pathway analyses were conducted using the DAVID bioinformatics resource (version 2021; http://david.ncifcrf.gov). The STRING database (version 12.0; https://cn.string-db.org/) was utilized to identify potential interacting partners of Zbp1, followed by Reactome pathway enrichment analysis to elucidate its functional context within biological pathways.

### Cell culture and treatments

MH-S murine AMs (ATCC CRL-2019) were cultured in RPMI-1640 medium (Gibco, USA) supplemented with 10% fetal bovine serum (FBS; VivaCell, China) under standard incubation conditions (37 ^∘^C, 5% CO_2_). To induce an inflammatory response, cells were exposed to LPS (Solarbio, China) at concentrations of 0.5 µg/mL or 1 µg/mL for 12 h. Following stimulation, cells were collected for further experimental procedures. To investigate the role of mitochondrial reactive oxygen species (mtROS), Mito-TEMPO (MT; MedChemExpress, China) was applied at a concentration of 100 µM, added 30 min prior to LPS treatment to inhibit mtROS accumulation.

### Lactate dehydrogenase (LDH) release detection

MH-S cells were cultured in 96-well plates and treated with LPS (Solarbio, China) at concentrations of 0.5 µg/mL or 1 µg/mL for 12 h. After stimulation, the supernatant was collected to assess LDH release using a commercial LDH cytotoxicity detection kit (Beyotime, China) following the manufacturer’s instructions. Absorbance was measured at 450 nm using a BioTek multi-mode microplate reader (USA).

### Cellular immunofluorescence

MH-S cells were stained with MitoTracker Red at 37 ^∘^C for 30 min in the dark. After incubation, cells were fixed with 4% paraformaldehyde, permeabilized with 0.1% Triton X-100 (Beyotime, China) for 20 min, and blocked with 5% bovine serum albumin (BSA; Solarbio, China) at room temperature for 30 min. Cells were then incubated overnight at 4 ^∘^C with a rabbit-derived primary antibody against murine Zbp1 (cat.#:13285-1-AP, Proteintech, China; dilution 1:100). Following washing, cells were treated with an FITC-labeled goat anti-rabbit IgG secondary antibody (cat.#:A0562, Beyotime, China; dilution 1:250) for 1 h in the dark. Nuclear staining was performed using DAPI (cat.#:C1006, Beyotime, China; dilution 1:500) for 10 min at room temperature. Confocal images were acquired using a laser scanning microscope (Nikon, Japan).

### mtROS detection

mtROS production in MH-S cells was evaluated using the fluorescent probe MitoSOX Red (5 µM). The cells were incubated with the dye at 37 ^∘^C for 30 min in the dark, followed by three washes with phosphate-buffered saline (PBS). Imaging was performed using a laser-scanning confocal microscope (Nikon, Japan), and fluorescence intensity was quantitatively analyzed by flow cytometry (BD Biosciences, USA).

### Assessment of mitochondrial membrane potential (Δ Ψm)

Mitochondrial membrane potential (Δ Ψm) was assessed using the JC-1 assay kit (Beyotime, China). MH-S cells were rinsed with PBS and incubated in JC-1 working solution at 37 ^∘^C for 30 min in the dark. Following incubation, unbound dye was removed by washing with pre-chilled staining buffer. The fluorescence signal ratio (aggregated red vs. monomeric green) was analyzed via fluorescence microscopy (Nikon, Japan) and quantified using a flow cytometer (BD Biosciences, USA).

### Detection of mitochondrial permeability transition pore (MPTP) opening

The status of the MPTP was evaluated using a commercial assay kit (Beyotime, China). MH-S cells were treated with Calcein-AM in combination with cobalt chloride to quench cytosolic fluorescence, followed by a 30-min incubation at 37 ^∘^C in the dark. After thorough washing, cellular fluorescence was observed under a fluorescence microscope (Nikon, Japan) and quantitatively measured by flow cytometry (BD Biosciences, USA).

### Measurement of adenosine triphosphate (ATP)

Cellular ATP levels were quantified using an enhanced luminescence-based ATP detection kit (Beyotime, China). After treatment with the lysis buffer provided in the kit, cell lysates were centrifuged to obtain the supernatant. A standard curve was created using serial dilutions of ATP standards, and the detection reagent was prepared as instructed. Luminescence signals were recorded with a multifunctional microplate reader (BioTek, USA). Protein content was assessed by measuring absorbance at 280 nm, and ATP levels were normalized to total protein concentration.

### Flow cytometry

MH-S cells were immunolabeled with anti-CD86 allophycocyanin (PE) and anti-CD206 phycoerythrin (APC) antibodies (eBioscience, USA) to characterize surface markers. The cells were incubated with these antibodies at 4 ^∘^C for 30 min in the dark, followed by washing with PBS. Subsequent analysis was conducted on a FACS Canto II flow cytometer (BD Biosciences, USA), with marker levels quantified by fluorescence intensity.

To evaluate necroptosis, the Calcein/PI Cell Viability/Cytotoxicity Assay Kit (Beyotime, China) was utilized. After washing twice with chilled PBS, cells were resuspended in 1× Binding Buffer and incubated with Calcein-AM and propidium iodide (PI) dyes for 30 min at room temperature in the dark. Immediately afterward, stained cells underwent flow cytometric analysis. To exclude alternative cell death pathways, Zbp1-knockdown MH-S cells were co-treated for 4 h with the pyroptosis inducer Nigericin (5 µg/mL; TargetMol, China), the apoptosis inducer Staurosporine (1 µM; TargetMol, China), or the ferroptosis inducer RSL3 (10 µM; TargetMol, China). Data processing was conducted using FlowJo software (Version 10.8.1).

### Quantitative real-time PCR (qRT-PCR)

Total RNA was isolated according to the manufacturer’s instructions using an RNA extraction kit (BioFLUX, China). Equivalent amounts of RNA were reverse transcribed into cDNA utilizing a reverse transcription kit (Accurate Biology, China). qRT-PCR was performed with SYBR qRT-PCR Premix (Accurate Biology, China) following the provided protocol. For mitochondrial RNA analysis, mitochondria were first purified from MH-S cells using a mitochondrial isolation kit (Beyotime, China), and RNA was then extracted to assess mitochondrial Zbp1 transcript levels. β-actin or 18S rRNA served as reference genes for normalization. Relative expression levels were calculated using the 2^−ΔΔCt^ method. Primer sequences employed in this study are detailed in Table S1.

The isolated mitochondrial fraction was diluted 100-fold for morphological examination. Following a 10-min staining with Janus green B, the mitochondria were observed under a light microscope. For protein analysis, the mitochondrial fraction was lysed using a mitochondrial lysis buffer to extract mitochondrial proteins. Subsequently, the mitochondrial marker COXIV (cat.#:250135, ZEN-BIO, China, dilution 1:1000) and the cytosolic marker GAPDH (cat.#:390035, ZEN-BIO, China, dilution 1:1000) were detected via Western blotting in both the mitochondrial and cytosolic fractions, respectively.

### Zbp1 siRNA transfection

Transfection was conducted using the EndoFection™ MAX transfection reagent (GeneCopoeia, China). Cells were seeded in 12-well plates, with each well receiving a transfection mixture comprising 5 µL of the transfection reagent and 1 µg of siRNA. Following transfection, cells were incubated for 36 h. The sequences of the siRNA primers are provided in Table S2.

### Western blotting

Total proteins were extracted using lysis buffer (KeyGEN Biotech, China) supplemented with 100 mM PMSF and a protease inhibitor cocktail to prevent degradation. Protein concentrations were determined by absorbance at 280 nm. Equal amounts of protein were loaded onto SDS-PAGE gels for electrophoretic separation, followed by transfer onto PVDF membranes (Millipore, USA). Membranes were blocked with 5% BSA for 1 h at room temperature and then incubated overnight at 4 ^∘^C with primary antibodies against translocase of outer mitochondrial membrane 20 (TOMM20) (cat.#:382451, ZEN-BIO, China, dilution 1:1000), ATP5a1 (cat.#:R381760, ZEN-BIO, China, dilution 1:1000), GAPDH (cat.#:390035, ZEN-BIO, China, dilution 1:1000), TFAM (cat.#:ab307302, Abcam, USA, dilution 1:1000), Zbp1 (cat.#:13285-1-AP, Proteintech, China, dilution 1:1000), MLKL (cat.#:66675-1-Ig, Proteintech, China, dilution 1:5000), RIPK3 (cat.#:68786-2-Ig, Proteintech, China, dilution 1:2000), phospho-MLKL (p-MLKL) (cat.#:37333, CST, USA, dilution 1:1000), and phospho-RIPK3 (p-RIPK3) (cat.#:91702, CST, USA, dilution 1:1000), with antibody dilutions as recommended by the manufacturers. After washing, membranes were treated with horseradish peroxidase (HRP)-conjugated goat anti-rabbit IgG (cat.#:SA00001-2, Proteintech, China, dilution 1:2000) for 1 h at room temperature. Protein bands were visualized using enhanced chemiluminescence reagents (ZEN-BIO, China) and captured by a Bio-Rad imaging system (Bio-Rad, USA). Band intensities were quantified using ImageJ software.

### Ethical statement

The research protocol was reviewed and approved by the Institutional Ethics Committee of the Children’s Hospital of Chongqing Medical University (Approval ID: CHCMU-IACUC20220804007).

### Statistical analysis

All experiments were conducted with at least three independent biological replicates. Statistical analyses and graphing were performed using GraphPad Prism version 9.0. Results are presented as mean ± standard error of the mean (SEM). Comparisons between groups employed Student’s *t*-test or one-way ANOVA, depending on the data structure. A *P* value of less than 0.05 was considered statistically significant.

## Results

### Transcriptomic profiling uncovers enhanced activation of Zbp1-mediated necroptotic pathways following LPS exposure

**Figure 1.**
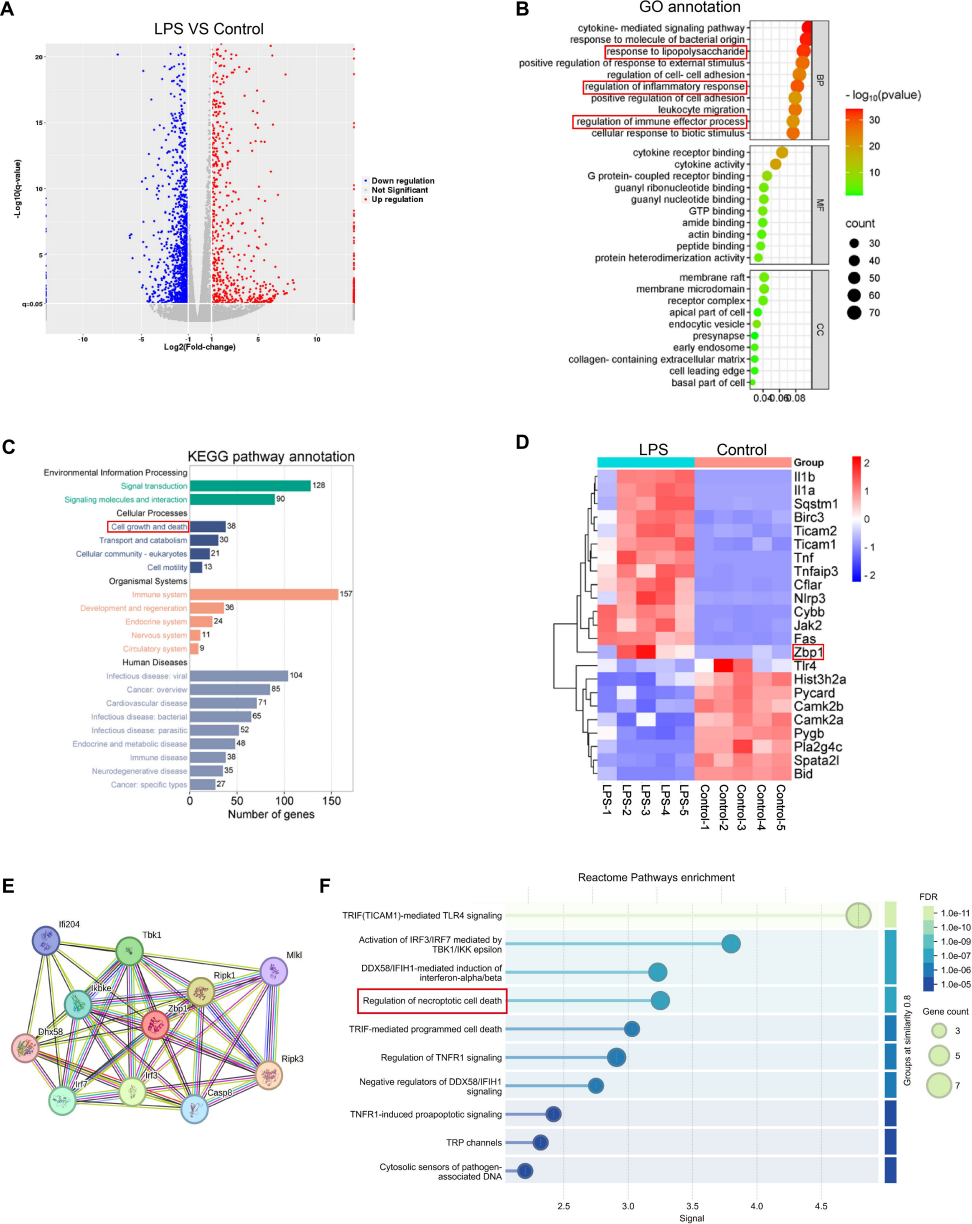
**Transcriptomic profiling of LPS-stimulated MH-S cells.** (A) A volcano plot illustrating the transcriptomic landscape of differentially expressed genes between LPS-stimulated MH-S cells and untreated control cells. (B) GO enrichment analysis highlighting the ten most significant biological processes associated with the differentially expressed genes. (C) KEGG pathway enrichment analysis of the top 100 differentially expressed genes, emphasizing key molecular pathways modulated by LPS stimulation. (D) A heatmap visualization of differentially expressed genes specifically associated with the necroptosis signaling pathway, showcasing distinct expression patterns. (E) Potential interacting partners of Zbp1 identified in the PPI network. (F) Reactome pathway enrichment analysis related to Zbp1 and its interacting partners. Abbreviations: LPS: Lipopolysaccharide; MH-S: Murine alveolar macrophage-like cell line; GO: Gene ontology; KEGG: Kyoto encyclopedia of genes and genomes; PPI: Protein–protein interaction.

To investigate the molecular pathways through which LPS induces gene expression alterations and necroptosis in MH-S cells, we conducted an extensive transcriptomic comparison between untreated control samples and LPS-treated cells (*n* ═ 5 per group). The differential expression analysis, depicted in a volcano plot (Figure 1A), identified numerous significantly regulated genes. Notably, Zbp1 exhibited marked upregulation (log_2_ fold change = 4.634, *P* ═ 0.000139), suggesting its potential involvement in the cellular response to LPS. GO enrichment analysis revealed that the top ten biological processes were predominantly related to cytokine-mediated signaling, cellular responses to LPS stimulation, and inflammatory regulation (Figure 1B). Furthermore, KEGG pathway analysis of the 100 most DEGs indicated significant enrichment in pathways related to cell proliferation and programmed cell death mechanisms (Figure 1C). Hierarchical clustering of necroptosis-associated genes demonstrated coordinated transcriptional modulation, with 14 genes, including Zbp1, showing increased expression, while 9 genes were downregulated (Figure 1D). Collectively, these findings imply a key role for Zbp1 in mediating necroptotic signaling induced by LPS in AMs. The protein–protein interaction (PPI) network identified several key proteins, including RIPK3, RIPK1, and MLKL, as potential interacting partners of Zbp1 (Figure 1E). Reactome pathway enrichment analysis further revealed a significant association of Zbp1 with the regulation of necroptosis (Figure 1F).

### LPS-induced activation of Zbp1-mediated necroptosis in MH-S cells

**Figure 2.**
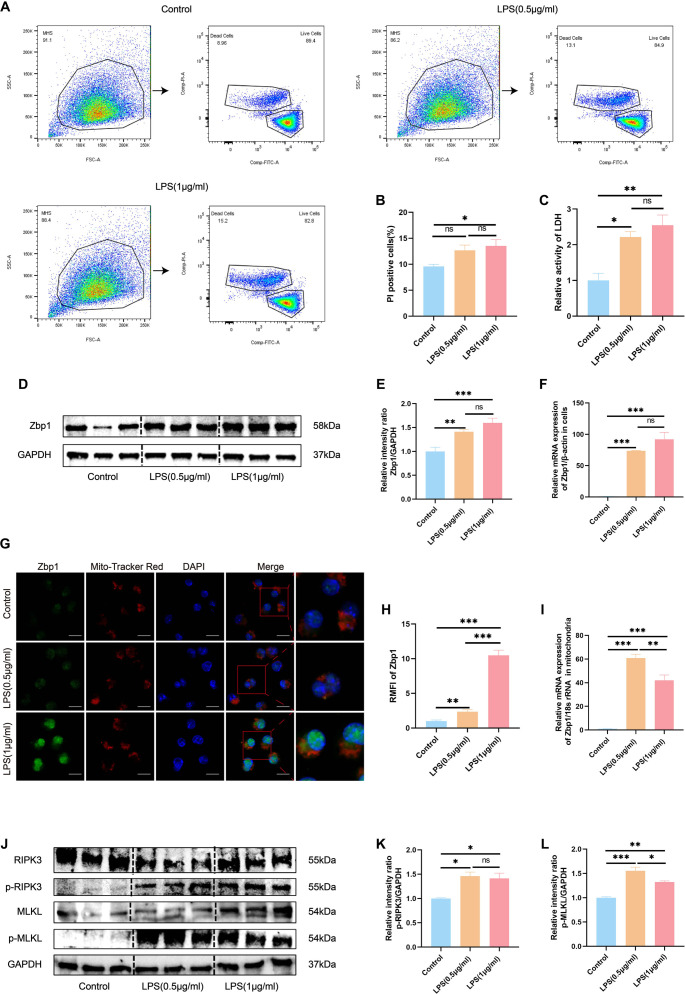
**LPS-induced activation of the Zbp1-mediated necroptosis in MH-S cells.** (A--B) Flow cytometric analysis employing Calcein/PI staining was conducted to evaluate cell viability and necroptosis, with results quantitatively analyzed. (C) Lactate dehydrogenase (LDH) enzymatic activity assays quantified necroptosis in MH-S cells. (D--E) Western blot analysis assessed Zbp1 protein expression levels, utilizing GAPDH as a loading control, with corresponding densitometric quantifications presented. (F) Quantitative real-time PCR (qRT-PCR) was performed to measure the relative mRNA expression levels of Zbp1 in MH-S cells following stimulation with various concentrations of LPS. (G--H) Confocal microscopy facilitated the visualization and quantitative analysis of Zbp1 colocalization with mitochondria, with a scale bar of 25 µm. (I) qRT-PCR was also employed to assess the relative mRNA levels of Zbp1 localized in the mitochondrial fraction of MH-S cells. (J) Western blot analysis evaluated the protein expression levels of RIPK3, p-RIPK3, MLKL, and p-MLKL in MH-S cells, again using GAPDH as a loading control. (K--L) Densitometric analysis of the Western blot results for p-RIPK3 and p-MLKL is presented. Data are expressed as mean ± SEM, *n* ≥ 3. Normality was assessed using the Shapiro-Wilk test. One-way ANOVA was performed, followed by Tukey's test for multiple comparisons. Statistical significance was defined as *P < 0.05, **P < 0.01, and ***P < 0.001; ns indicates not significant. Abbreviations: LPS: Lipopolysaccharide; MH-S: Murine alveolar macrophage-like cell line; PI: Propidium iodide; LDH: Lactate dehydrogenase; GAPDH: Glyceraldehyde-3-phosphate dehydrogenase; RIPK3: Receptor-interacting protein kinase 3; p-RIPK3: Phosphorylated receptor-interacting protein kinase 3; MLKL: Mixed lineage kinase domain-like protein; p-MLKL: Phosphorylated mixed lineage kinase domain-like protein; SEM: Standard error of the mean; ANOVA: Analysis of variance; qRT-PCR: Quantitative real-time PCR.

Cell death via necroptosis was assessed through Calcein/PI staining, LDH release assays, and analysis of the Zbp1/RIPK3/MLKL signaling axis. Flow cytometric analysis revealed a dose-dependent increase in the percentage of PI-positive MH-S cells following treatment with LPS at concentrations of 0.5 µg/mL and 1 µg/mL (Figure 2A and 2B). Correspondingly, LDH activity was significantly elevated in the culture media (Figure 2C). To investigate Zbp1 regulation, quantitative PCR and Western blotting were performed, demonstrating substantial increases in Zbp1 expression at both the transcript (Figure 2F) and protein levels (Figure 2D and 2E) post-LPS exposure. Given the critical role of mitochondria in programmed cell death pathways [18], we further examined Zbp1’s intracellular localization. Immunofluorescence imaging with MitoTracker Red indicated an increase in Zbp1 expression, showing clear mitochondrial colocalization in LPS-stimulated cells (Figure 2G and 2H). This observation was validated by qPCR analysis of isolated mitochondrial fractions, which exhibited elevated Zbp1 mRNA following LPS treatment (Figure 2I). The isolated mitochondria were confirmed using Janus green B staining and Western blotting, as depicted in Figure S1A and S1B. Moreover, Western blot results indicated significant increases in p-RIPK3 and p-MLKL proteins post-LPS stimulation (Figure 2J–2L). To further elucidate the role of Zbp1 in LPS-induced necroptosis in MH-S cells, Zbp1 was specifically knocked down using siRNA (knockdown efficiency validated in Figure S2A–S2C; the siZbp1-1 sequence was selected for subsequent experiments). Calcein/PI staining revealed that Zbp1 knockdown significantly reduced the proportion of dead cells after LPS stimulation (Figure S2D and S2E). qPCR confirmed the reduction in Zbp1 mRNA levels following knockdown in MH-S cells, indicating that the knockdown cells did not respond to LPS challenge (Figure S2F). The protein levels of Zbp1, p-RIPK3, and p-MLKL were assessed via Western blotting, revealing that Zbp1 knockdown led to decreased expression of p-RIPK3 and p-MLKL after LPS stimulation (Figure S2G–S2J). Consistent with its role in necroptosis, the death of Zbp1-knockdown cells was significantly reduced following LPS challenge. In contrast, these cells remained susceptible to cell death induced by Nigericin (a pyroptosis inducer), Staurosporine (an apoptosis inducer), and RSL3 (a ferroptosis inducer), as evidenced by a significant increase in cell death (Figure S2K and S2L). Collectively, these data substantiate that Zbp1 functions as a specific mediator of necroptosis, while alternative cell death pathways operate independently of Zbp1. These findings suggest that LPS induces necroptosis through Zbp1 activation, mitochondrial targeting, and subsequent engagement of necroptotic downstream effectors in MH-S cells.

### LPS-induced mitochondrial dysfunction in MH-S cells

**Figure 3.**
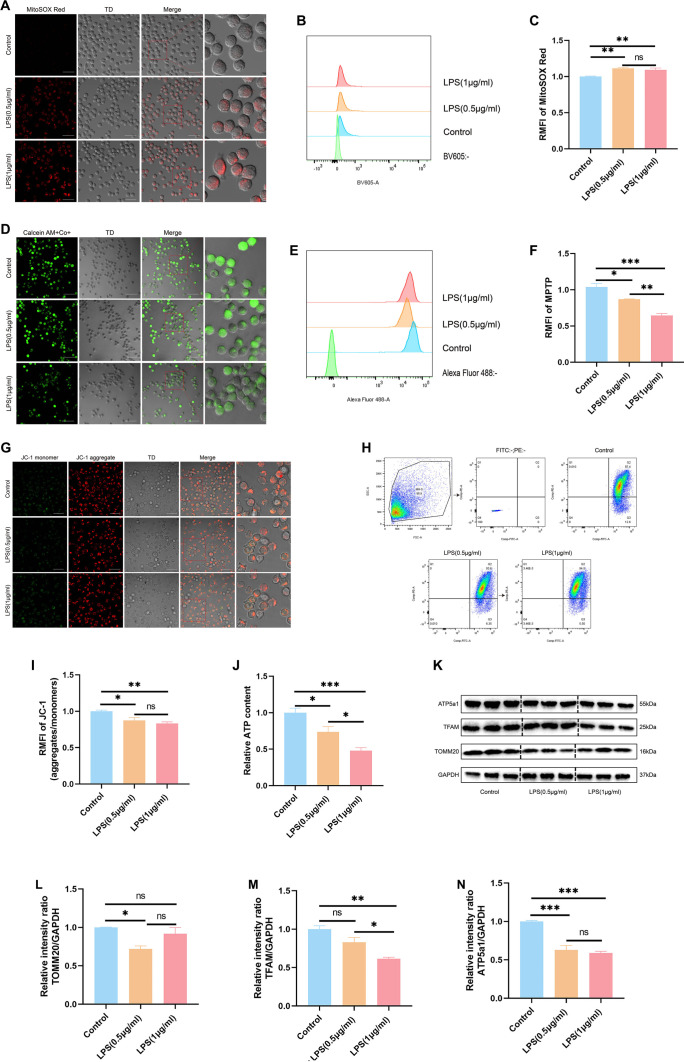
**LPS-induced mitochondrial dysfunction in MH-S cells.** (A) Confocal microscopy image of MH-S cells stained with MitoSOX Red, highlighting mitochondrial superoxide production. Scale bar = 25 µm; (B and C) Flow cytometry analysis and corresponding quantification of MitoSOX Red fluorescence intensity; (D) Confocal microscopy image of MH-S cells stained with MPTP, reflecting MPTP opening. Scale bar = 25 µm; (E and F) Flow cytometry analysis and corresponding quantification of MPTP fluorescence intensity; (G) Confocal microscopy image of MH-S cells stained with JC-1, demonstrating Δ Ψm. Scale bar = 25 µm; (H and I) Flow cytometry analysis and statistical results of JC-1 fluorescence intensity; (J) ATP content measurement in MH-S cells; (K) Western blot analysis of TOMM20, TFAM, and ATP5A1 protein expression in MH-S cells, with GAPDH as the loading control; (L–N) Statistical quantification of TOMM20, TFAM, and ATP5a1 protein expression from western blot data. Data are presented as mean ± SEM, *n* ≥ 3. Normality was assessed using the Shapiro–Wilk test. For one-way ANOVA, Tukey’s test was subsequently used for multiple comparisons. Statistical significance was denoted as *P < 0.05, **P < 0.01, and ***P < 0.001, while ns indicated non-significant differences. Abbreviations: LPS: Lipopolysaccharide; MH-S: Murine alveolar macrophage-like cell line; MPTP: Mitochondrial permeability transition pore; Δ Ψm: Mitochondrial membrane potential; TOMM20: Translocase of outer mitochondrial membrane 20; TFAM: Mitochondrial transcription factor A; ATP5A1: ATP synthase F1 subunit alpha; GAPDH: Glyceraldehyde-3-phosphate dehydrogenase; SEM: Standard error of the mean; ANOVA: Analysis of variance.

To investigate how LPS affects mitochondrial function in MH-S cells, we evaluated several indicators, including mtROS generation, MPTP opening, and Δ Ψm. Both confocal microscopy and flow cytometry analyses demonstrated a significant increase in mtROS levels in cells treated with LPS compared to untreated controls (Figure 3A–3C). Additionally, LPS exposure facilitated MPTP opening (Figure 3D–3F) and resulted in a marked reduction in Δ Ψm (Figure 3G–3I).

Assessment of mitochondrial bioenergetic status further revealed a substantial decrease in ATP synthesis following LPS stimulation (Figure 3J). Correspondingly, the expression of essential mitochondrial proteins, such as TOMM20, TFAM, and ATP5a1, was notably diminished after LPS treatment (Figure 3K–3N). Together, these findings provide strong evidence that LPS induces significant mitochondrial dysfunction in MH-S cells.

### LPS-induced inflammatory response in MH-S cells

**Figure 4.**
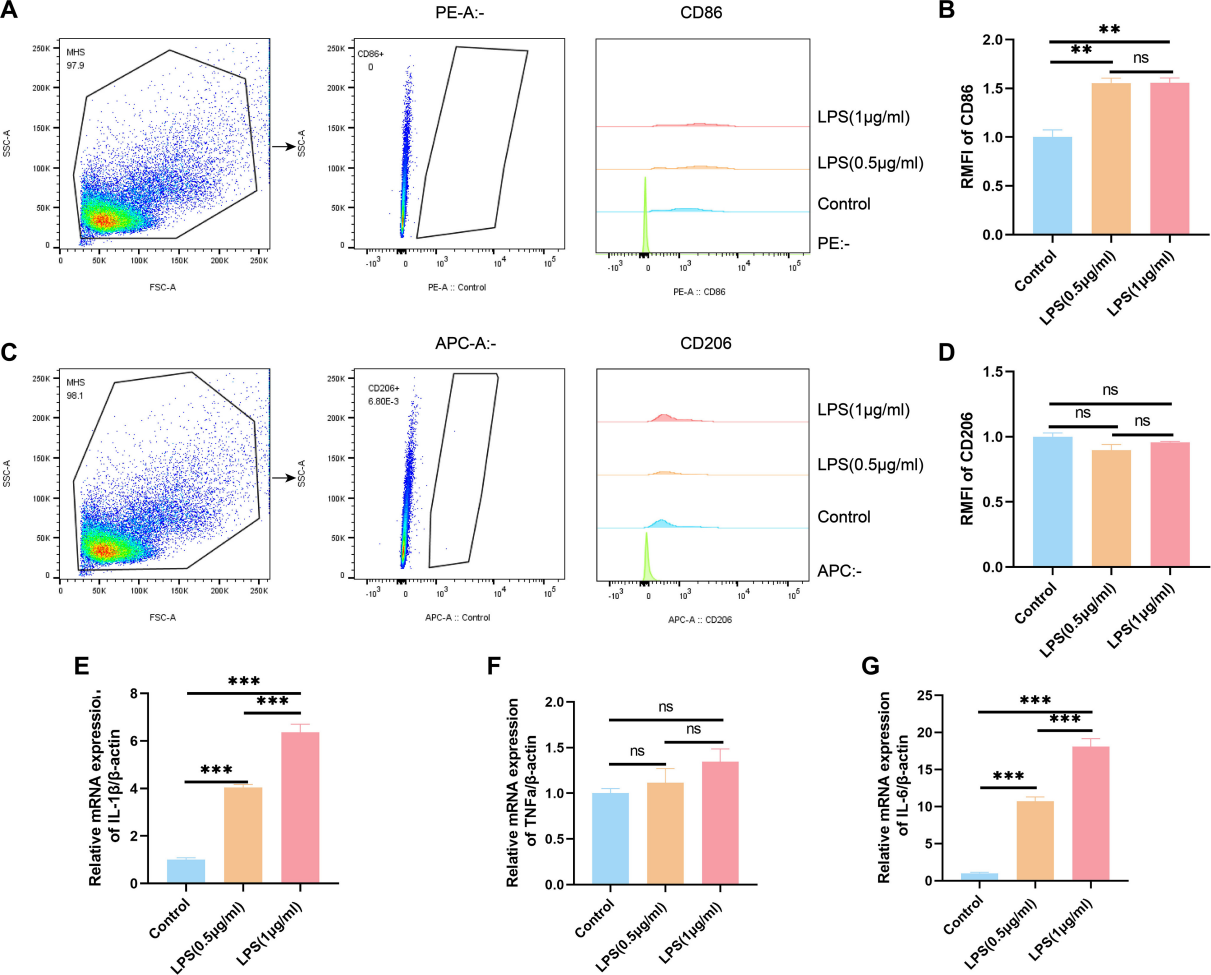
**LPS-induced inflammatory response in MH-S cells.** (A) Flow cytometric analysis of CD86 expression in MH-S cells following LPS stimulation. (B) Quantitative analysis of the RMFI for CD86 in LPS-stimulated MH-S cells. (C) Flow cytometric analysis of CD206 expression in MH-S cells after LPS stimulation. (D) Quantitative analysis of the RMFI for CD206 in LPS-stimulated MH-S cells. (E--G) qRT-PCR analysis of relative mRNA expression levels of the pro-inflammatory cytokines IL-1β, TNF-α, and IL-6 in LPS-stimulated MH-S cells. Data are presented as mean ± SEM, with *n* ≥ 3. Normality was assessed using the Shapiro-Wilk test. One-way ANOVA was performed, followed by Tukey's test for multiple comparisons. Statistical significance was denoted as **P < 0.01 and ***P < 0.001, while ns indicated non-significant differences. Abbreviations: LPS: Lipopolysaccharide; MH-S: Murine alveolar macrophage-like cell line; RMFI: Relative mean fluorescence intensity; TNF-α: Tumor necrosis factor alpha; SEM: Standard error of the mean; ANOVA: Analysis of variance; qRT-PCR: Quantitative real-time PCR.

Macrophages exhibit significant plasticity, allowing them to modify their activation states in response to inflammatory signals to support various immune and homeostatic functions [19, 20]. To investigate the response of MH-S cells to LPS stimulation, we utilized flow cytometry to measure levels of CD86 and CD206, surface markers indicative of M1 and M2 macrophage subsets, respectively. Concurrently, qPCR was employed to evaluate the expression of key pro-inflammatory cytokines such as IL-1β, TNF-α, and IL-6, thereby defining the inflammatory phenotype of the cells. Flow cytometric data revealed a pronounced increase in CD86-positive cells after LPS exposure, suggesting polarization toward the M1, or classically activated, macrophage phenotype (Figure 4A–4D). Meanwhile, qPCR results indicated significant elevations in IL-1β and IL-6 mRNA levels following LPS treatment (Figure 4E–4G). Collectively, these findings demonstrate that LPS stimulation enhances inflammatory signaling in MH-S cells and drives their differentiation toward a pro-inflammatory M1 state, underscoring LPS’s role as a key inducer of macrophage activation.

### mtROS depletion restores mitochondrial function in MH-S cells

To investigate the role of mtROS in mitochondrial damage, MH-S cells were pre-incubated with MT, a specific mitochondrial ROS scavenger, prior to LPS stimulation. Based on preliminary tests, 1 µg/mL of LPS was selected for subsequent experiments.

**Figure 5.**
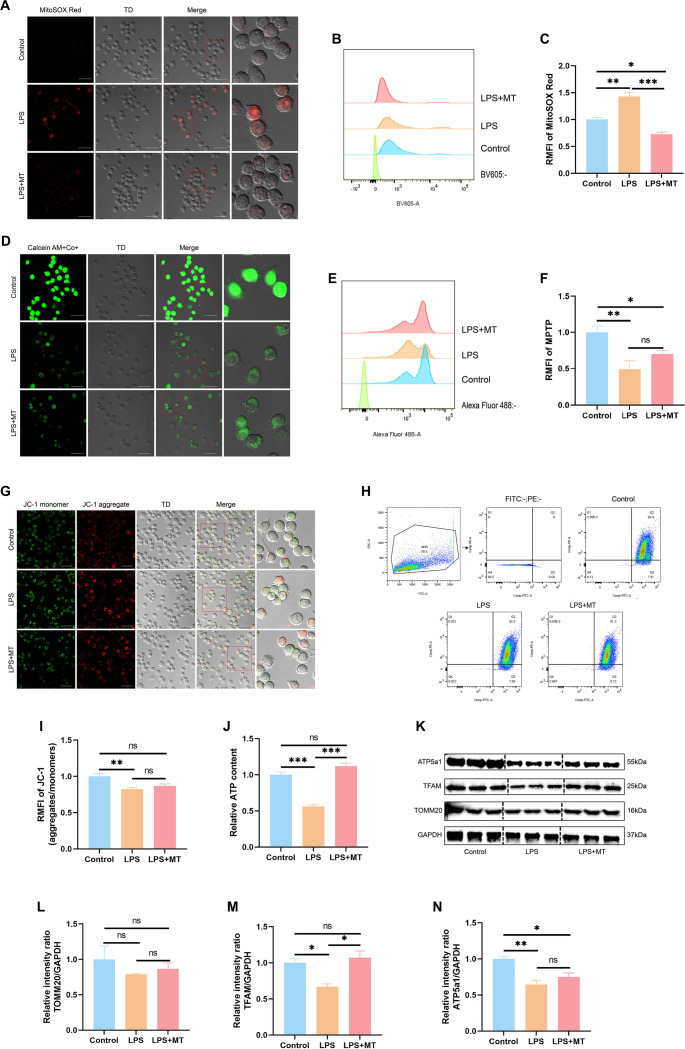
**Depletion of mtROS restores mitochondrial function.** (A) Confocal microscopy image of MH-S cells stained with MitoSOX Red. Scale bar ═ 25 µm. (B--C) Flow cytometric analysis and statistical results of MitoSOX Red staining in MH-S cells. (D) Confocal microscopy image of MH-S cells stained with MPTP. Scale bar ═ 25 µm. (E--F) Flow cytometric analysis and statistical results of MPTP staining in MH-S cells. (G) Confocal microscopy image of MH-S cells stained with JC-1. Scale bar ═ 25 µm. (H--I) Flow cytometric analysis and statistical results of JC-1 staining in MH-S cells. (J) Measurement of ATP content in MH-S cells. (K) Western blot analysis to quantify protein expression levels of TOMM20, TFAM, and ATP5a1 in MH-S cells, with GAPDH serving as a loading control. (L--N) Statistical results from the Western blot analysis of TOMM20, TFAM, and ATP5a1. Data are presented as mean ± SEM, *n* ≥ 3. Normality was assessed using the Shapiro-Wilk test, and one-way ANOVA was followed by Tukey's test for multiple comparisons. Statistical significance was denoted as *P < 0.05, **P < 0.01, and ***P < 0.001, while ns indicated non-significant differences. Abbreviations: mtROS: Mitochondrial reactive oxygen species; MH-S: Murine alveolar macrophage-like cell line; MPTP: Mitochondrial permeability transition pore; JC-1: Mitochondrial membrane potential probe; TOMM20: Translocase of outer mitochondrial membrane 20; TFAM: Mitochondrial transcription factor A; ATP5a1: ATP synthase F1 subunit alpha; GAPDH: Glyceraldehyde-3-phosphate dehydrogenase; SEM: Standard error of the mean; ANOVA: Analysis of variance; ATP: Adenosine triphosphate.

The levels of mtROS, MPTP opening, and Δ Ψm were quantitatively evaluated using confocal imaging, flow cytometry, and JC-1 staining assays. MT pretreatment significantly attenuated the increase in mtROS induced by LPS in MH-S cells (Figure 5A–5C). Furthermore, it inhibited MPTP opening (Figure 5D–5F) and helped maintain Δ Ψm, which was otherwise diminished after LPS exposure (Figure 5G–5I). Intracellular ATP content was notably higher in cells pretreated with MT compared to those treated solely with LPS (Figure 5J). Additionally, TFAM protein levels were significantly elevated following MT treatment relative to the LPS group (Figure 5K–5N). Collectively, these findings demonstrate that MT-mediated clearance of mtROS effectively mitigates LPS-induced mitochondrial dysfunction in MH-S cells.

### mtROS depletion mitigates Zbp1-mediated necroptosis in MH-S cells

**Figure 6.**
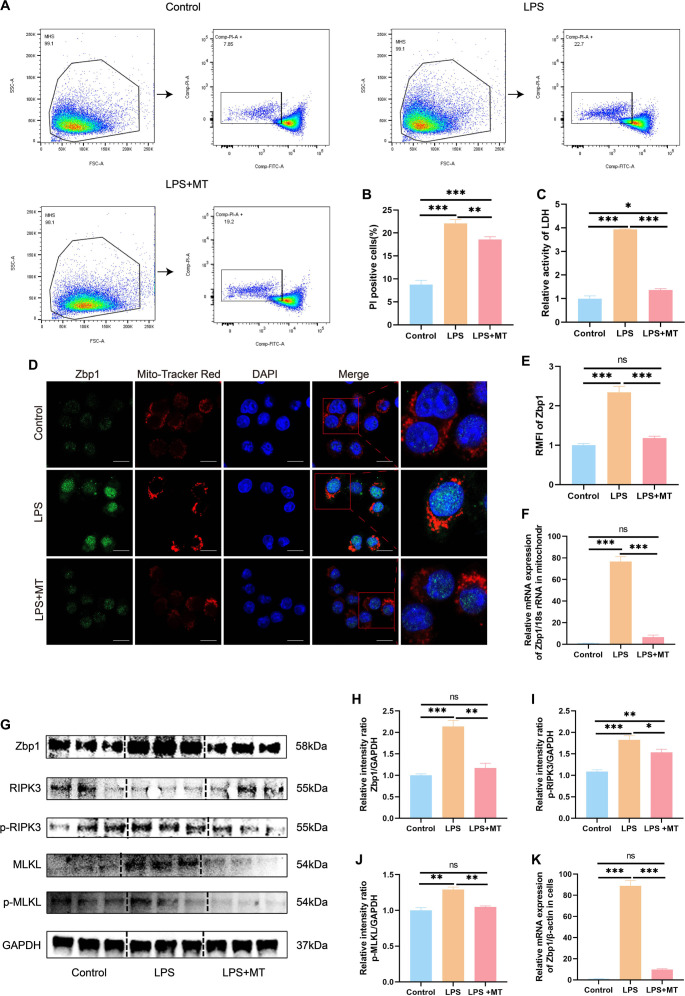
**Depletion of mtROS attenuates Zbp1-mediated necroptosis.** (A--B) Flow cytometric analysis and corresponding statistical results of Calcein/PI staining in MH-S cells. (C) Statistical analysis of LDH enzymatic activity assays in MH-S cells. (D--E) Confocal microscopy visualization of Zbp1 colocalization with mitochondria, accompanied by the corresponding statistical representation on the right. Scale bar ═ 25 µm. (F) qRT-PCR was employed to assess the relative mRNA levels of Zbp1 in the mitochondria of MH-S cells. (G) Western blot analysis was conducted to quantify the protein expression levels of Zbp1, RIPK3, p-RIPK3, MLKL, and p-MLKL in MH-S cells, with GAPDH serving as the internal loading control. (H--J) Statistical analysis of the western blot results for Zbp1, p-RIPK3, and p-MLKL. (K) qRT-PCR was again utilized to determine the relative mRNA levels of Zbp1 in MH-S cells. Data are presented as mean ± SEM, with *n* ≥ 3. Normality was assessed using the Shapiro-Wilk test. A one-way ANOVA was performed, followed by Tukey's test for multiple comparisons. Statistical significance was denoted as *P < 0.05, **P < 0.01, and ***P < 0.001, while ns indicated non-significant differences. Abbreviations: mtROS: Mitochondrial reactive oxygen species; MH-S: Murine alveolar macrophage-like cell line; PI: Propidium iodide; LDH: Lactate dehydrogenase; RIPK3: Receptor-interacting protein kinase 3; p-RIPK3: Phosphorylated receptor-interacting protein kinase 3; MLKL: Mixed lineage kinase domain-like protein; p-MLKL: Phosphorylated mixed lineage kinase domain-like protein; GAPDH: Glyceraldehyde-3-phosphate dehydrogenase; SEM: Standard error of the mean; ANOVA: Analysis of variance; qRT-PCR: Quantitative real-time PCR.

To delineate the relationship between mitochondrial dysfunction and necroptosis, we investigated whether eliminating mtROS could modulate Zbp1-mediated necroptosis. Flow cytometry demonstrated a significant reduction in PI-positive cell populations in the LPS + MT group compared to the LPS-only treated group (Figure 6A and 6B). Consistently, MT pretreatment resulted in decreased LDH levels in the culture supernatant (Figure 6C). To rule out potential cytostatic or off-target effects of MT, we compared the proportion of PI-positive cells using Calcein/PI staining between the untreated control and MT-only treated MH-S cells. The results indicated no significant difference in cell death between the two groups (Figure S3A and S3B). Additionally, Zbp1 expression was markedly suppressed, and its colocalization with mitochondria was reduced following MT administration (Figure 6D and 6E). Mitochondrial RNA analysis via qPCR further revealed that clearance of mtROS led to significant downregulation of Zbp1 transcripts within the mitochondria of LPS-exposed MH-S cells (Figure 6F).

To further elucidate the mechanistic basis, Western blot assays were performed to assess the expression of necroptosis-associated markers, including Zbp1, RIPK3, p-RIPK3, MLKL, and p-MLKL. MT pretreatment significantly suppressed the protein levels of Zbp1 as well as p-RIPK3 and p-MLKL compared to LPS treatment alone (Figure 6G–6J). Additionally, in MH-S cells, pretreatment with MT also reduced the mRNA levels of Zbp1 (Figure 6K). Collectively, these findings indicate that neutralization of mtROS attenuates Zbp1-dependent necroptotic signaling in MH-S cells.

### mtROS depletion attenuates inflammatory responses in MH-S cells

This study investigates the effects of reducing mtROS on macrophage polarization and inflammatory signaling. We employed flow cytometry to assess the expression of surface markers related to M1 and M2 phenotypes and performed qPCR to evaluate cytokine gene expression. This integrated approach aimed to elucidate the influence of mitochondrial restoration and the inhibition of Zbp1-driven necroptosis on the inflammatory profile.

**Figure 7.**
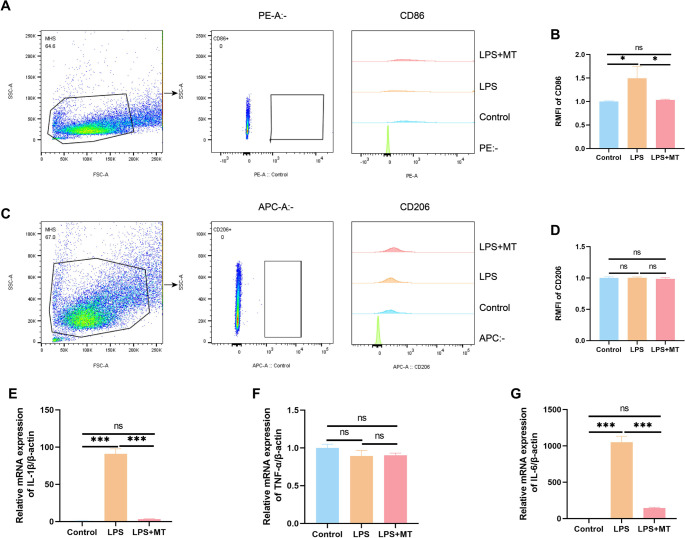
**Depletion of mtROS attenuates inflammatory responses in MH-S cells.** (A) Representative flow cytometry plots illustrating CD86 expression levels in MH-S cells. (B) Quantitative analysis of the RMFI of CD86 in MH-S cells. (C) Representative flow cytometry plots showing CD86 expression levels in MH-S cells. (D) Quantitative analysis of the RMFI of CD206 in MH-S cells. (E--G) qRT-PCR was employed to assess the relative mRNA expression levels of IL-1β, TNF-α, and IL-6 in MH-S cells. Data are presented as mean ± SEM, with *n* ≥ 3. Normality was evaluated using the Shapiro-Wilk test. One-way ANOVA was performed, followed by Tukey's test for multiple comparisons. Statistical significance was denoted as *P < 0.05 and ***P < 0.001, while ns indicated non-significant differences. Abbreviations: mtROS: Mitochondrial reactive oxygen species; MH-S: Murine alveolar macrophage-like cell line; RMFI: Relative mean fluorescence intensity; TNF-α: Tumor necrosis factor alpha; IL-6: Interleukin-6; SEM: Standard error of the mean; ANOVA: Analysis of variance; qRT-PCR: Quantitative real-time PCR.

Flow cytometry results indicated that treatment with MT in conjunction with LPS resulted in a significant reduction in CD86 fluorescence intensity, suggesting a suppression of M1-like macrophage activation (Figure 7A–7D). Additionally, transcriptional analysis revealed decreased mRNA levels of the inflammatory cytokines IL-1β and IL-6 in this cohort (Figure 7E–7G). Collectively, these findings imply that neutralizing mtROS can diminish inflammatory activation in MH-S cells by shifting macrophage polarization away from the M1 phenotype and reducing cytokine expression.

## Discussion

**Figure 8.**
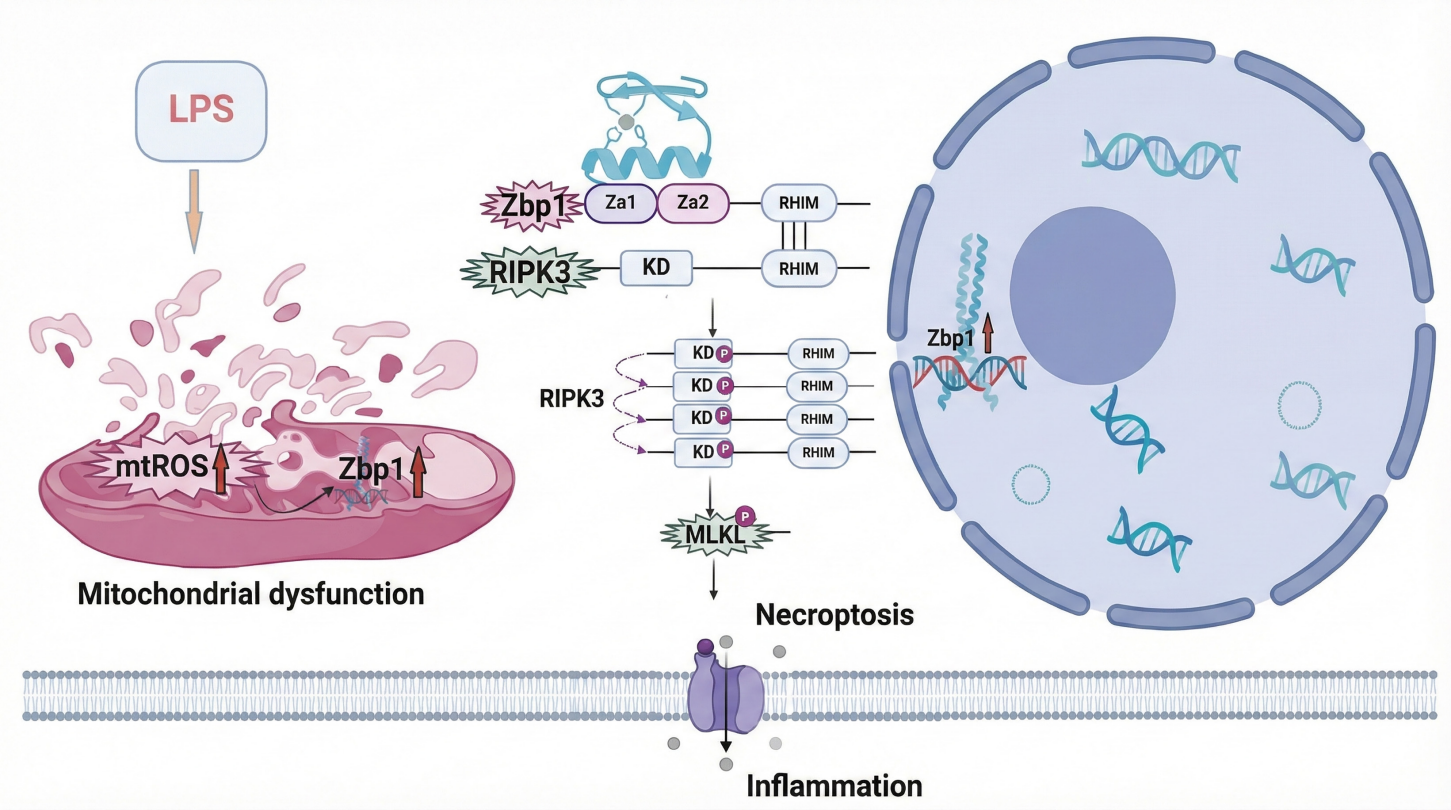
**Schematic illustration of Zbp1-mediated necroptosis induced by mitochondrial dysfunction and its role in inflammatory responses in alveolar macrophages during ALI.** This schematic illustrates the pathological mechanisms that drive inflammatory responses in alveolar macrophages during ALI. Mitochondrial dysfunction leads to the accumulation of ROS, which subsequently activate the Zbp1/RIPK3/MLKL necroptosis pathway. The execution of necroptosis exacerbates inflammation by promoting the release of pro-inflammatory cytokines, thereby amplifying the inflammatory cascade and contributing to the progression of ALI. Abbreviations: ALI: Acute lung injury; ROS: Reactive oxygen species; RIPK3: Receptor-interacting protein kinase 3; MLKL: Mixed lineage kinase domain-like protein.

In this study, we identified mitochondrial impairment as a critical upstream factor contributing to Zbp1-dependent necroptosis in AMs during ALI, thereby exacerbating the inflammatory response. Importantly, the depletion of mtROS was shown to restore mitochondrial integrity, suppress Zbp1-mediated necroptotic signaling, and inhibit the polarization of macrophages toward the pro-inflammatory M1 phenotype, ultimately alleviating lung inflammation (Figure 8).

Excessive mtROS disrupts oxidative phosphorylation by impairing the electron transport chain and increasing mitochondrial membrane permeability, which exacerbates mitochondrial dysfunction and promotes inflammation [21–23]. In our study, the clearance of mtROS restored mitochondrial membrane potential, limited MPTP opening, and enhanced ATP production. Furthermore, key proteins associated with mitochondrial integrity exhibited partial recovery, indicating that mtROS elimination mitigates LPS-induced mitochondrial damage. Notably, mtROS scavenging also reduced mitochondrial Zbp1 accumulation and inhibited the downstream phosphorylation of RIPK3 and MLKL, underscoring mtROS as a crucial upstream factor in Zbp1-driven necroptosis. Additionally, levels of pro-inflammatory cytokines associated with the M1 phenotype were significantly diminished, suggesting that mitochondrial dysfunction promotes necroptosis and inflammatory signaling, at least in part through mtROS-mediated Zbp1 activation.

Zbp1, initially recognized as a cytosolic sensor of Z-form nucleic acids via its Zα domains, has emerged as a key mediator in cellular stress pathways, including those triggered by oxidative stress [24–26]. Our immunofluorescence data demonstrated that LPS exposure elevated Zbp1 levels not only in the cytoplasm but also prominently within the nucleus and mitochondria, with significant colocalization in mitochondrial regions. Further experimental validation is required to confirm the mitochondrial localization of Zbp1. Studies utilizing co-immunoprecipitation or other complementary methods will be essential to definitively establish its mitochondrial presence and identify its direct interaction partners.

The downstream signaling cascade is initiated when Zbp1 acts as a scaffold to facilitate the recruitment and activation of RIPK3, which subsequently phosphorylates MLKL, the key effector that executes necroptosis [27–30]. Previous research has shown that Zbp1 can trigger RIPK3 activation within the nucleus, leading to MLKL phosphorylation and subsequent nuclear integrity disruption, resulting in the cytoplasmic release of nuclear contents [31, 32]. Consistent with these observations, our ALI animal model demonstrated that AMs exhibited characteristics of Zbp1-dependent necroptotic activity (refer to Supplementary Materials). Furthermore, LPS exposure promoted mtROS accumulation alongside increased levels of Zbp1, p-RIPK3, and p-MLKL, suggesting a close mechanistic relationship between oxidative stress and necroptotic signaling. Notably, pharmacological clearance of mtROS significantly reduced the expression of these necroptosis markers, reinforcing the concept that mtROS is a critical upstream signal driving Zbp1-mediated necroptosis in AMs. However, while the role of the Zbp1/p-RIPK3 pathway in ALI mice has been preliminarily validated in this study, the impact of MT on this pathway *in vivo* warrants further investigation. Future studies utilizing Zbp1 knockout mouse models will be necessary to confirm the *in vivo* function and elucidate the precise mechanisms of the Zbp1/p-RIPK3 pathway.

Overall, this study reveals novel insights into the upstream mechanisms by which mitochondrial impairment and mtROS initiate Zbp1-dependent necroptosis in the context of ALI. Our results underscore the pivotal role of Zbp1 as a molecular integrator connecting oxidative stress, mitochondrial damage, necroptotic signaling, and inflammation. These findings also raise several unresolved questions. For instance, it remains to be determined whether mtROS-driven Zbp1 activation results from direct oxidative modifications of the protein or through stress-induced Z-RNA formation. Additionally, the functional significance of Zbp1 translocation between mitochondria and the nucleus is not yet well understood. Future investigations should explore upstream modulatory pathways—such as mitochondrial DNA leakage, inflammasome dynamics, or activation of stress-responsive kinases—that may influence Zbp1 expression and necroptotic signaling. Furthermore, exploring pharmacological inhibitors or gene-editing strategies targeting the mtROS-Zbp1 axis may provide promising therapeutic avenues for treating ALI and other inflammatory lung disorders.

## Conclusion

Necroptosis and mitochondrial dysfunction are known contributors to inflammation, with LPS recognized as a trigger for these processes in various contexts. However, the specific coupling of LPS-induced mitochondrial damage in AMs to necroptosis and its amplification of inflammation has not been clearly defined. Here, we demonstrate that in AMs, LPS-induced mtROS serves as an upstream signal activating Zbp1, leading to programmed necroptosis. We further establish that this macrophage-specific necroptotic event is a key driver of the inflammatory cascade and tissue injury in our model. Therefore, our study provides a novel, cell-type-specific mechanistic link between mitochondrial injury and inflammation via necroptosis, refining the understanding of ALI pathogenesis.

In summary, we demonstrate that mitochondrial dysfunction in AMs—triggered by LPS and mediated through mtROS-dependent Zbp1 activation—acts as a key driver of inflammatory processes in ALI. Our findings suggest that modulation of mitochondrial homeostasis and interference with Zbp1-mediated necroptosis in macrophages may represent viable therapeutic strategies for mitigating inflammation and preventing tissue damage in ALI. This work lays a mechanistic foundation for the development of targeted interventions and could help refine current treatment strategies for inflammatory lung injury.

## Supplemental data

Supplemental data are available at the following link: https://www.bjbms.org/ojs/index.php/bjbms/article/view/13046/4032.

## Data Availability

The datasets used and/or analyzed during the current study are available from the corresponding author on reasonable request.
